# Absence of Platelet Phenotype in Mice Lacking the Motor Protein Myosin Va

**DOI:** 10.1371/journal.pone.0053239

**Published:** 2013-01-18

**Authors:** Matthew T. Harper, Marion T. J. van den Bosch, Ingeborg Hers, Alastair W. Poole

**Affiliations:** School of Physiology and Pharmacology, University of Bristol, Bristol, United Kingdom; Royal College of Surgeons, Ireland

## Abstract

**Background:**

The motor protein myosin Va plays an important role in the trafficking of intracellular vesicles. Mutation of the *Myo5a* gene causes Griscelli syndrome type 1 in humans and the *dilute* phenotype in mice, which are both characterised by pigment dilution and neurological defects as a result of impaired vesicle transport in melanocytes and neuroendocrine cells. The role of myosin Va in platelets is currently unknown. Rab27 has been shown to be associated with myosin Va cargo vesicles and is known to be important in platelet dense granule biogenesis and secretion, a crucial event in thrombus formation. Therefore, we hypothesised that myosin Va may regulate granule secretion or formation in platelets.

**Methodology/Principal Findings:**

Platelet function was studied *in vitro* using a novel *Myo5a* gene deletion mouse model. *Myo5a* ^−/−^ platelets were devoid of myosin Va, as determined by immunoblotting, and exhibited normal expression of surface markers. We assessed dense granule, α-granule and lysosomal secretion, integrin α_IIb_β_3_ activation, Ca^2+^ signalling, and spreading on fibrinogen in response to collagen-related peptide or the PAR4 agonist, AYPGKF in washed mouse platelets lacking myosin Va or wild-type platelets. Surprisingly, *Myo5a* ^−/−^ platelets showed no significant functional defects in these responses, or in the numbers of dense and α-granules expressed.

**Conclusion:**

Despite the importance of myosin Va in vesicle transport in other cells, our data demonstrate this motor protein has no non-redundant role in the secretion of dense and α-granules or other functional responses in platelets.

## Introduction

The unconventional, non-muscle, class V myosins play an important role in the transport of intracellular vesicles along actin filaments to membrane docking sites [1]. The isoform myosin Va has been shown to be crucial in the trafficking of melanosomes in melanocytes [2] and in the secretion of granules in neuroendocrine cells [3]. Mutations in the *Myo5a* gene lead to Griscelli syndrome type 1 in humans, a rare inherited autosomal recessive disorder characterised by hypopigmentation and neurological impairment [4]. In mice, myosin Va mutations result in the *dilute* phenotype with a lighter coat colour and lethal neurological defects [5].

A variety of proteins involved in the regulation of granule transport has been described to interact with myosin Va. In melanocytes, the cargo-carrying C-terminal tail of myosin Va binds to the exophilin melanophilin [6], [7] which in turn interacts with Rab27 [8], a GTP-binding protein of the Ras superfamily. Knockout of Rab27a/b in mice and Griscelli syndrome type 2 in humans, caused by mutation of the Rab27a gene, both show platelet defects resulting in increased bleeding times and a reduction in the number of dense granules, indicating that Rab27 is a key player in platelet dense granule biogenesis and secretion [9]–[12].

The secretion of intracellular granules from platelets is essential in the process of thrombosis. Upon activation, platelets release a wide array of mediators from their dense and α-granules [13]. Dense granules contain pro-aggregating factors, which sustain and enhance initial platelet responses. In addition to molecules involved in thrombus formation, α-granules also store a range of proteins and receptors involved in other patho-physiological processes, such as inflammation. As Rab27 is known to be associated with myosin Va cargo vesicles and myosin Va is highly expressed in both human and mouse platelets [14], it is of great interest to determine the role of this motor protein in platelet granule secretion and formation.

In the present study, we used a novel targeted *Myo5a* gene deletion mouse model, which is non-lethal and shows no overt neurological phenotype. As shown by immunoblotting, myosin Va was undetectable in platelets from *Myo5a* ^−/−^ mice. Levels of surface proteins and granule counts were normal in *Myo5a* ^−/−^ platelets. Contrary to our expectations, loss of myosin Va resulted in normal agonist-induced dense and α-granule secretion and unchanged numbers of dense and α-granules expressed. Furthermore, integrin α_IIb_β_3_ activation, Ca^2+^ signalling, and spreading on fibrinogen were not affected in *Myo5*a^−/−^ platelets. These results establish that myosin Va is not required for most platelet responses, including dense and α-granule secretion.

## Methods

### Materials

The myosin Va antibody (#3402) was from Cell Signaling Technology (Danvers, MA, USA). The myosin Vb antibody (18), the myosin Vc antibody (Y-19), and the GAPDH antibody (6C5) were obtained from Santa Cruz Biotechnology (Santa Cruz, CA, USA). The myosin VI antibody (ABT42) was from Millipore (Temecula, CA, USA). Horseradish peroxidase (HRP)-conjugated donkey anti-rabbit, anti-mouse and anti-goat secondary antibodies were from Jackson ImmunoResearch Laboratories (Newmarket, UK). FITC-P-selectin and PE-JON/A antibodies were from Emfret Analytics (Eibelstadt, Germany). FITC-LAMP1 (1D4B) antibody was from Abcam (Cambridge, UK). NuPAGE LDS sample buffer was obtained from Invitrogen (Carlsbad, CA, USA). 10× blocking buffer and TRITC-phalloidin were from Sigma-Aldrich (Poole, UK). Fura-PE3 was from Teflabs (Austin, TX, USA). Luciferin-luciferase was from Chronolog (Labmedics, Stockport, UK). AYPGKF-NH_2_ (PAR4 activating peptide) was from Bachem (Weil-am-Rhein, Germany). CRP (cross-linked collagen-related peptide) was synthesized by Prof Richard Farndale (Department of Biochemistry, University of Cambridge, UK).

### Mouse platelet preparation

A colony of *Myo5a^−/−^* mice was obtained from the Wellcome Trust Sanger Institute (Cambridge, UK), which were mated with C57/Bl6j mice (Charles River, UK) to generate *Myo5a^+/−^*. These mice were crossed to generate *Myo5a^−/−^* mice and littermate *Myo5a^+/+^* mice, which were used as control (wild-type, WT). Use of mouse platelets was approved by local research committee at the University of Bristol, UK, and mice were bred for this purpose under UK Home Office Licence PPL30/2908 held by Alastair W. Poole. Blood was drawn and washed platelets were prepared as described previously [15]. In brief, blood was drawn by cardiac puncture under terminal anesthesia into sodium citrate (4%; 1∶10 v/v). Blood was diluted with modified Tyrode's-HEPES buffer (134 mM NaCl, 2.9 mM KCl, 20 mM HEPES, 5 mM glucose, and 1 mM MgCl_2_, pH 7.3) and centrifuged at 180 *g* for 6 minutes at room temperature. Platelet-rich plasma was removed, and platelets were isolated by centrifugation at 550 *g* for 6 minutes in the presence of PGE_1_ (140 nM) and apyrase (0.02 U/ml). Pelleted platelets were resuspended to the required density in modified Tyrode's-HEPES buffer and rested for 30 minutes at 37°C in the presence of 0.02 U/ml apyrase prior to stimulation. No indomethacin or other cyclooxygenase inhibitors were added during platelet preparation. Unless otherwise indicated, 1 mM CaCl_2_ was added immediately prior to stimulation.

### Electrophoresis and Western blotting

Washed platelets (2×10^8^/mL) were lysed in NuPAGE LDS sample buffer which was supplemented with 50 mM dithiothreitol. Samples were separated by electrophoresis on 6% Bis-Tris polyacrylamide gels. Proteins were then transferred to polyvinylidene difluoride membranes which were blocked with 1× blocking buffer and probed with specific primary and HRP-conjugated secondary antibodies. Proteins were detected using ECL reagents.

### Subcellular morphology

Subcellular morphology of WT and *Myo5a* ^−/−^ platelets was analysed by transmission electron microscopy (TEM). Platelet-rich plasma was collected and spun at 590 *g* for 5 minutes. Supernatant was removed from the platelet pellet, and the pellet was fixed in 2.5% glutaraldehyde in 0.1 M phosphate buffer (PB) (pH 7.4). The pellet was washed in PB and then incubated in 1% osmium tetroxide in PB for 30 minutes. After washing in PB and deionized water, the pellet was incubated in 3% uranyl acetate in deionized water for 30 minutes. After washing with deionized water, the pellet was dehydrated in a graded series of increasing amounts of ethanol (70%, 80%, 90%, 96%, 100%, and 100%, with each step lasting for 10 minutes). After removal of the 100% ethanol, the pellet was incubated with pure Epon for 2 hours at room temperature. Thereafter, the Epon was replaced with fresh Epon, and this was hardened overnight in a 60°C oven. Ultrathin counterstained sections were imaged on a Philips CM100 equipped with a side-mount MegaView III camera (Olympus Soft Imaging Solutions).

To determine the dense-granule and α-granule content, total numbers of granules in equivalent-sized fields of view were counted. For each genotype, 10 randomly chosen fields of view were examined. All microscopic images were taken at the same magnification, and the number of cells per field of view was equivalent between WT and *Myo5a^−/−^* preparations. The number of dense granules and α-granules is expressed as the mean number per platelet slice. This approach allows the number of each granule type to be compared between WT and *Myo5a^−/−^* platelets, although since individual thin sections of platelets are imaged, the number of granules seen is substantially fewer than the total number of granules in individual platelets.

### Dense granule secretion

The release of ATP from dense granule release was assessed luminometrically, as previously described [15]. Briefly, platelets were incubated with Chrono-Lume luciferase-luciferin reagent before stimulation with the indicated concentration of agonist. ATP secretion was measured as an increase in luminescence.

### Integrin α_IIb_β_3_ activation and α-granule secretion

Washed platelets (5×10^7^/mL; 32 µL) were incubated fluorescein isothiocyanate (FITC)-labelled anti-P-selectin antibody, phycoerythrin (PE)-labelled JON/A antibody (4 µL of each), and agonist (4 µL; 1∶10 dilution) for 10 min under non-stirring conditions. Platelets were then fixed with paraformaldehyde (2%). Two-colour analysis was conducted by flow cytometry on a FACSCalibur flow cytometer (BD Biosciences), using CellQuest version 3.1f software (BD Biosciences). The platelet population was identified by forward and side scatter profile.

To assess the timecourse of α-granule secretion, platelets were stimulated for the indicated time with AYPGKF (300 µM) prior to fixation. FITC-labelled anti-P-selectin was added for the final 30 s of stimulation.

### Lysosome secretion

Washed platelets (5×10^7^/mL; 36 µL) were incubated FITC-labelled anti-LAMP1 antibody (4 µL), and agonist (4 µL; 1∶10 dilution) for 10 min under non-stirring conditions. Platelets were then fixed with paraformaldehyde (2%). FITC fluorescence was analysed by FACS analysis as above.

### Ca^2+^ signalling

Changes in cytosolic [Ca^2+^]_i_ were measured by spectrofluorometry in washed suspensions of platelets (1×10^8^/mL) loaded with the Ca^2+^-senstive dye, Fura-PE3, as described previously [16]. Platelets were stimulated at 37°C with continuous stirring. Fura-PE3 was excited alternately at 340 nm and 380 nm, and fluorescence emission detected at 510 nm. Fluorescence signals were corrected for autofluorescence and calibrated in terms of [Ca^2+^]_i_ as previously described [16]. Ionomycin (1 µM) was added in the presence of the calcium chelator EGTA (1 mM). Alternatively, platelets were stimulated with the indicated concentrations of CRP in the presence of 1 mM CaCl_2_.

### Platelet spreading

Coverslips were coated with fibrinogen (100 µg/ml). Washed platelets (2×10^7^/mL) were dispensed onto the coverslip for 1 hr. Where indicated, platelets were stimulated with AYPGKF (300 µM) for 1 min prior to adhesion. Adherent platelets were fixed with paraformaldehyde (4%), permeabilised with Triton X-100, and stained with TRITC-phalloidin. Adhesion and spreading of platelets was observed by fluorescence microscopy using a cooled CCD camera attached to a Leica DM IRB inverted epifluorescence microscope with a 63× objective. Ten images were taken in different random parts of the coverslip area and analysed using ImageJ software. Platelets were scored as being completely unspread with no filopodia (“no spreading”), having filopodia, having both filopodia and partial lamellipodia (“some spreading”), or having a full lamellipodium (“full spreading”) and expressed as a proportion of the total number of platelets in the image. The proportions of each morphology in each of the ten images were then averaged. This analysis was performed separately on platelets from 3 WT mice and 3 *Myo5a^−/−^* mice.

### Statistics

Where presented, mean data are given ± SEM. Statistical significance was determined by 2-way ANOVA with Bonferroni post-test, performing using Prism 4.0 (GraphPad Software). P<0.05 was considered significant.

## Results and Discussion

### Selective depletion of myosin Va from mouse platelets

**Figure 1:**
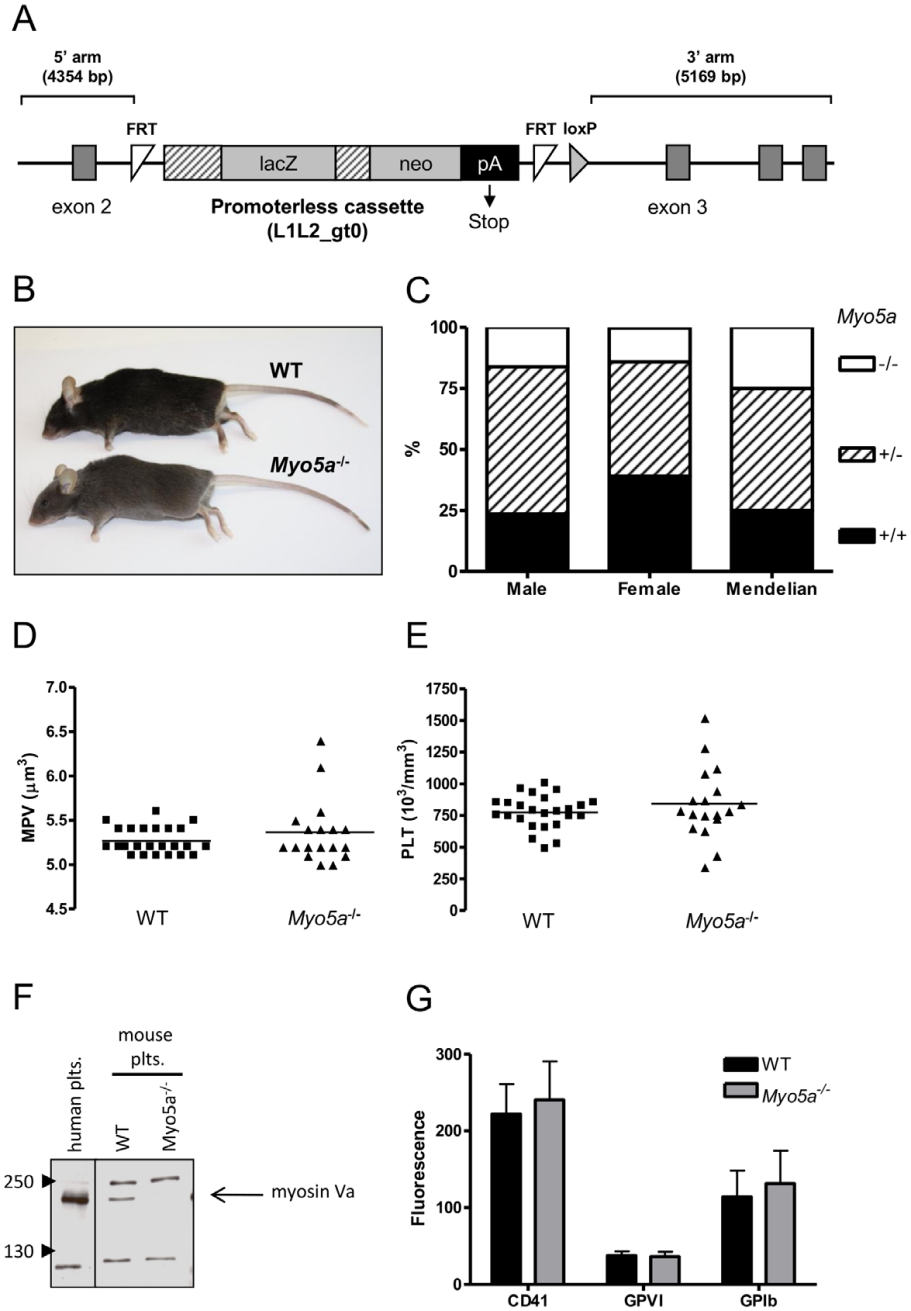
Phenotyping of *Myo5a* ^−/−^ mice and platelets. (A) Schematic of the targeting vector used to disrupt the *Myo5a* genomic locus between exons 2 and 3. The cassette contains the lacZ reporter gene, the neomycin (neo) resistance gene, and a polyadenylate (pA) sequence which causes transcription termination. Flp recombination target (FRT) sites are included on either side of the vector along with a loxP site at the 3′ end. In (B), the difference in coat pigmentation of *Myo5a* ^−/−^ mice compared to wild-type (WT) is shown. (C) Offspring ratios of knockouts (−/−), heterozygotes (+/−) and wild-types (+/+) from heterozygote breeding pairs were compared from 162 mice. Blood platelet count (D) and mean platelet volume (E) were measured in *Myo5a* ^−/−^ and wild-type (WT) mice (n = 18 and 25, respectively). (F) Immunoblots showing the expression of myosin Va protein in lysates from human, wild-type (WT) mouse and *Myo5a* ^−/−^ mouse platelets. Note the absence of a detectable myosin Va band in the knockout platelets. (G) Platelet surface levels of the indicated proteins were analysed by flow cytometry. Data (mean +/− SEM, n = 4–7) are mean fluorescence intensity levels.

To evaluate the function of myosin Va in platelets, *Myo5a^−/−^* mice were obtained from Wellcome Trust Sangar Institute (Cambridge, UK), in which the gene encoding this protein (*Myo5a*) had been disrupted by homologous recombination in embryonic stem cells, and the cells were used to generate gene-targeted mice by standard techniques (Fig. 1A). *Myo5a* ^−/−^ mice were viable and healthy, but showed a lighter grey coat colour compared to wild-type controls (Fig. 1B), similar to the hypopigmentation which is seen in the *dilute* mice. This suggests that our mice are likely to have a similar defect in Myo5a-dependent melanosome trafficking to *dilute* mice [5]. Mice were not obtained in their expected Mendelian ratios (Fig. 1C). Of 130 offspring genotyped, 48 were wild-type (37.5%), 63 were heterozygous (49.2%) and only 17 were *Myo5a* ^−/−^ (13.3%). Low generation of *Myo5a* ^−/−^ mice was seen in both male and female offspring. *Myo5a^−/−^* mice exhibited normal platelet count and mean platelet volume (Fig. 1D and E). Other haematological parameters, specifically the red blood cell count, haematocrit, mean corpuscular volume and white blood cell count, were also within the normal range (data not shown).

As demonstrated by immunoblotting, myosin Va (207 kDa) was not detected in *Myo5a* ^−/−^ platelets, whereas it was expressed in wild-type mouse platelets as well as in human platelets (Fig. 1F). *Myo5a* ^−/−^ platelets had normal levels of surface-expressed CD41, glycoprotein (GP) VI and GPIb (Fig. 1G). Thus, *Myo5a* ^−/−^ platelets provided us an ideal opportunity to study platelet function in the selective absence of myosin Va.

### Platelet granule biogenesis and secretion are unaffected by loss of myosin Va

**Figure 2:**
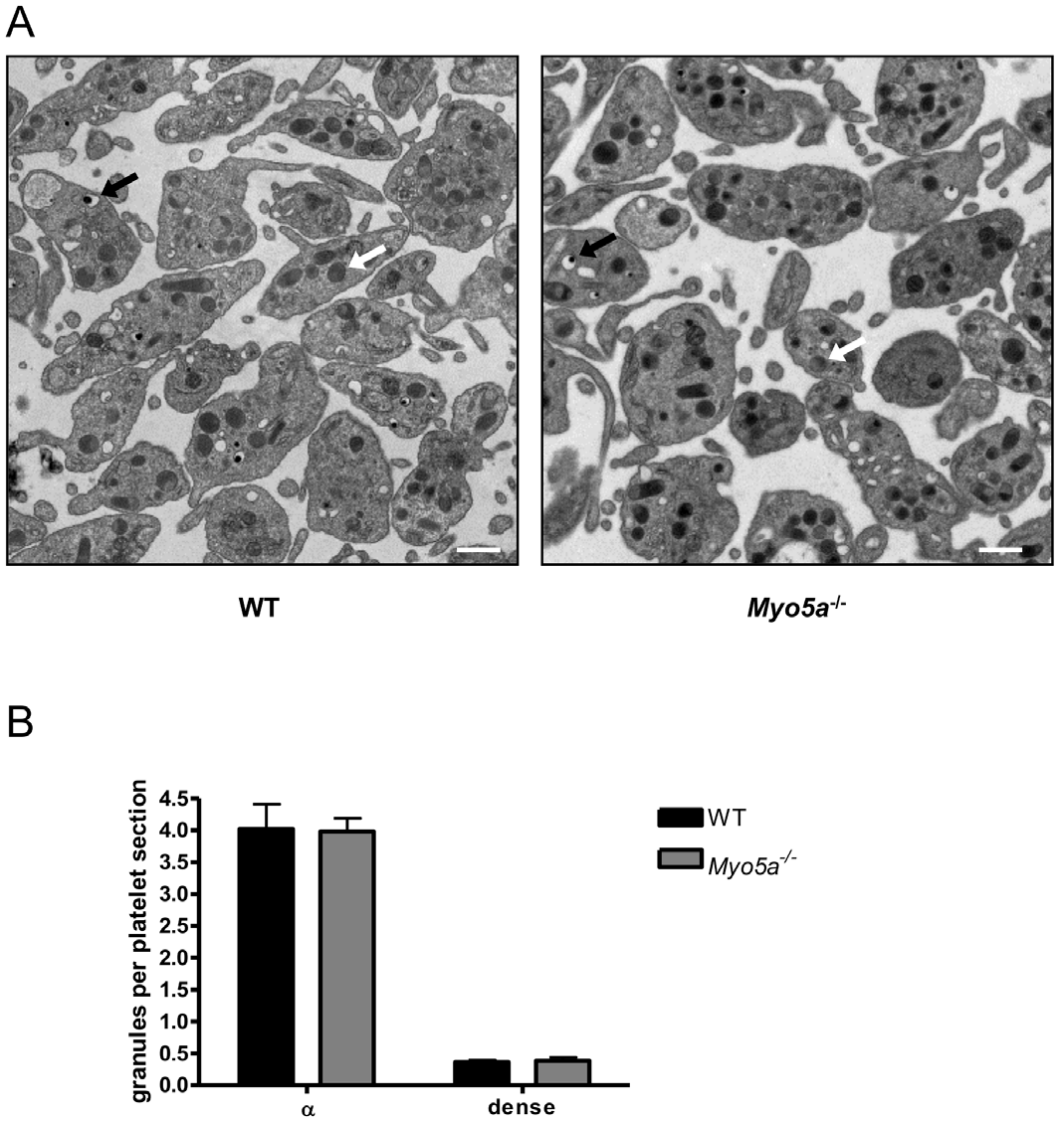
*Myo5a* ^−/−^ platelets have normal subcellular morphology. (A) TEM images (4800×, scale bar: 1 µM) show representative images of WT and *Myo5a* ^−/−^ mouse platelets (n = 4). Black arrows: dense granules. White arrows: α-granules. (B) Platelet dense and α-granules were quantified per field of view (10 fields of view per preparation) and data (mean +/− SEM, n = 4) are shown as the number of granules per platelet visible in the section. This number is for comparison between genotypes only, as granules above or below the thin section plane will not be visible, and so the number will be an underestimate of the total granule count per platelet.

Since Rab27 regulates dense granule formation and secretion in platelets [10] and has been shown to interact with myosin Va in melanocytes [8], we investigated whether myosin Va is also involved in platelet dense granule formation and secretion. Subcellular morphology of platelets from WT and *Myo5a* ^−/−^ mice was examined by TEM (Fig. 2A), and visible granules counted (and normalized to the number of platelets in the field of view; Fig. 2B). Both dense and α-granule counts in each thin section were similar to wild-type, suggesting that myosin Va is not involved in platelet granule biogenesis.

**Figure 3:**
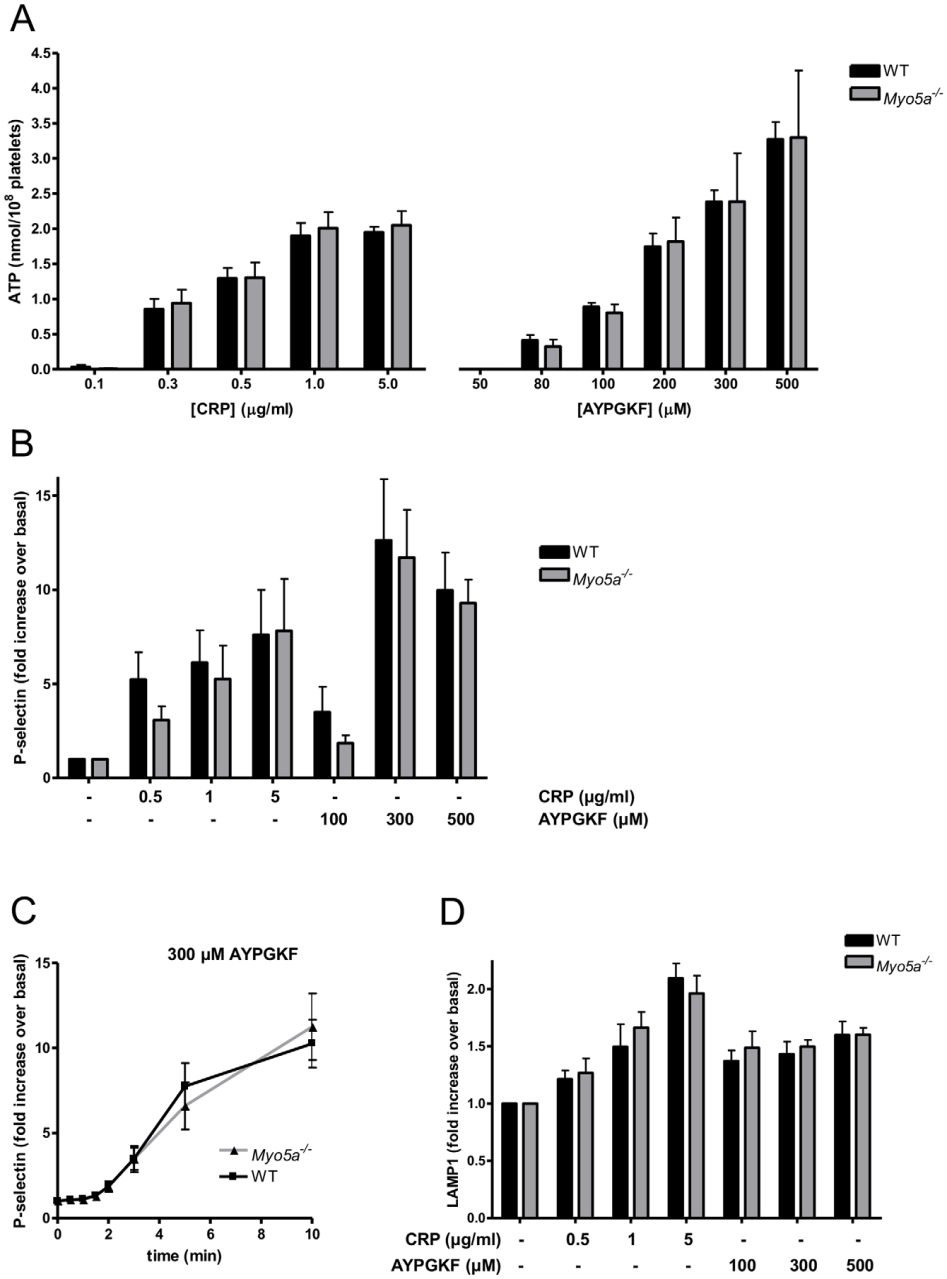
Loss of myosin Va does not affect platelet dense, α-granule or lysosome secretion. Wild-type and *Myo5a* ^−/−^ platelets were stimulated with the indicated concentrations of collagen-related peptide (CRP) and the PAR4 agonist AYPGKF. (A) ATP release from dense granules was assessed luminometrically. Data (mean +/− SEM, n = 4–7) are levels of released ATP. (B) P-selectin expression as a result of α-granule secretion was measured by flow cytometry, after agonist stimulation for 10 min. Data (mean +/− SEM, n = 5–7) are shown as fold increase over basal. (C) Time course of P-selectin expression induced by AYPGKF (300 µM). Data (mean +/− SEM, n = 4) are levels of FITC fluorescence intensity. (D) Lysosome secretion, as assessed by LAMP1 flow cytometry, was determined after agonist stimulation for 10 min. Data (mean +/− SEM, n = 4) are shown as fold increase over basal.

Dense granule secretion of ATP, monitored by luminometry, was stimulated by a range of concentrations of the GPVI agonist, collagen-related peptide (CRP) or the thrombin receptor PAR4 agonist, AYPGKF. However, no difference in ATP secretion was observed between wild-type and *Myo5a* ^−/−^ platelets at any concentration tested (Fig. 3A). This indicates that myosin Va is not required for the secretion of dense granule content.

Next, we addressed whether myosin Va has a role in α-granule secretion. By flow cytometric analysis, P-selectin expression on the platelet surface was assessed. P-selectin surface expression induced by various concentrations of CRP and AYPGKF was not significantly affected in *Myo5a* ^−/−^ platelets compared to wild-type (Fig. 3B), suggesting that myosin Va is not required also for α-granule secretion in platelets. In addition, analysis of the time course of α-granule secretion indicated that the rate of P-selectin expression was not different between wild-type and *Myo5a^−/−^* platelets (Fig. 3C).

Finally, we investigated whether myosin Va regulates lysosome secretion by assessing agonist-induced surface expression of LAMP1, which correlates with lysosomal enzyme release. Platelet activation induced an increase in surface LAMP1. This was not different between WT and *Myo5a^−/−^* platelets, indicating that lysosome secretion is not affected by loss of myosin Va. Taken together, these data show that myosin Va has no non-redundant role in granule secretion in mouse platelets.

There are several possible explanations for these data. It is possible that myosin Va genuinely plays no role in granule secretion in platelets, despite a role for Rab27. This could reflect the small size of the platelet, and its extensive plasma membrane-associated target membrane system, the open canalicular system. This is comprised of multiple invaginations of the membrane, forming target sites for fusion of exocytotic vesicles throughout the cell. Effectively, this may mean that the majority of secretory vesicles may already be in a primed and docked position, and that there is no need for myosin-dependent trafficking. It might however be assumed that vesicles require trafficking to platelets within the megakaryocyte, and therefore that myosins may be required for this step. However, data from Fig. 2A and 2B show that these platelets contain normal numbers of both α-granules and dense granules and therefore that myosin Va expression is also not required for normal trafficking and packaging of secretory granules into platelets at the level of the megakaryocyte. It is likely that, for this role, myosin II is the predominant motor protein involved [17], [18].

**Figure 4:**
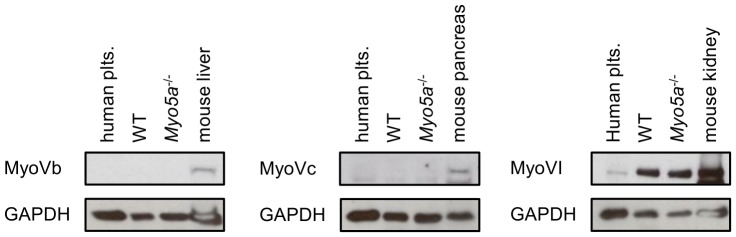
No enhanced expression of myosin Vb, Vc and VI in *Myo5a* ^−/−^ platelets. Immunoblots showing the expression of myosin Vb, Vc, and VI in lysates from human, wild-type mouse (WT) and *Myo5a* ^−/−^ mouse platelets. Lysates from mouse liver, pancreas or kidney were used as positive controls for myosin Vb, Vc, and VI, respectively. GAPDH served as loading control. Images shown are representative of three independent experiments.

On the other hand, it is also possible that other related myosins are expressed or over-expressed in the *Myo5a^−/−^* platelet, and that redundancy of function with these myosins is such that there is no observable phenotype in the single *Myo5a^−/−^* mouse. Myosin Vb is involved actin-dependent vesicle transport and plasma membrane recycling in various cells, including neurons and oocytes [19]–[21]. Myosin Vc localises with membrane compartments involved in transferrin trafficking in epithelial cells [22] and has been shown to function as a molecular motor driving secretory granule transport in lacrimal gland acinar cells and the MCF-7 cell line [23], [24]. To examine the expression of myosin Vb, Vc, and VI, we immunoblotted for these proteins in human, wild-type, and *Myo5a* ^−/−^ platelets (Fig. 4). Myosin Vb and Vc could not be detected in any platelet sample, suggesting that the absence of a platelet defect in myosin Va-deficient mice is not a consequence of normal expression or compensatory upregulation of myosin Vb or Vc. The absence of these proteins is consistent with mRNA analysis of human and mouse platelets [14].

Recently, it has been found that myosin VI regulates fusion pores formed between secretory vesicles and the plasma membrane [25]. In contrast to myosin Vb and Vc, myosin VI was present in human, wild-type and *Myo5a* ^−/−^ platelets. There was no upregulation of myosin VI detected in myosin Va-deficient platelets, suggesting that its expression does not change in the absence of myosin Va. However, the presence of myosin VI in platelets, and its recently reported role in exocytosis, provides the possibility that this protein is involved in platelet granule secretion, and that there may be redundancy of function between this motor protein and myosin Va in the control of granule secretion. This may be more important in mouse platelets than in human platelets, as mRNA analysis indicates a greater abundance of *myo6* mRNA in mouse platelets [14]. The possible role of myosin VI in mouse platelet exocytosis deserves further attention.

### *Myo5a* ^−/−^ platelets show normal integrin α_IIb_β_3_ activation, Ca^2+^ signalling and spreading

**Figure 5:**
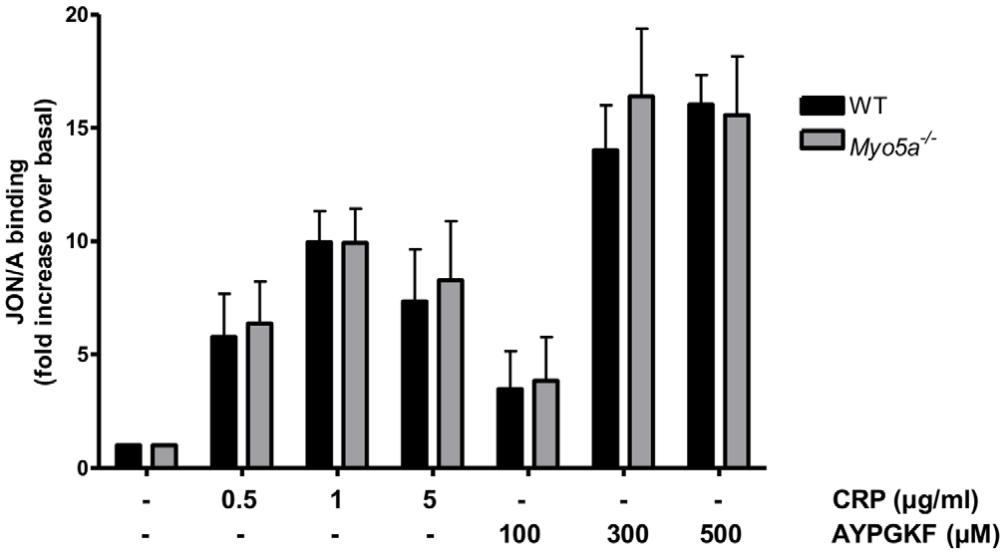
No difference in integrin α_IIb_β_3_ activation in myosin Va-deficient platelets. Wild-type and *Myo5a* ^−/−^ mouse platelets were stimulated for 10 min with the indicated concentrations of collagen-related peptide (CRP) and the PAR4 agonist AYPGKF. JON/A binding to the activated form of integrin α_IIb_β_3_ was measured by flow cytometry. Data (mean +/− SEM, n = 5) are shown as fold increase over basal.

It was important also to investigate whether myosin Va plays a role in other platelet responses. Integrin α_IIb_β_3_ activation was assessed by using the activation-specific antibody, Jon/A. CRP or AYPGKF induced integrin α_IIb_β_3_ activation in wild-type and *Myo5a* ^−/−^ platelets with no significant differences over a range of concentrations of agonists (Fig. 5).

**Figure 6:**
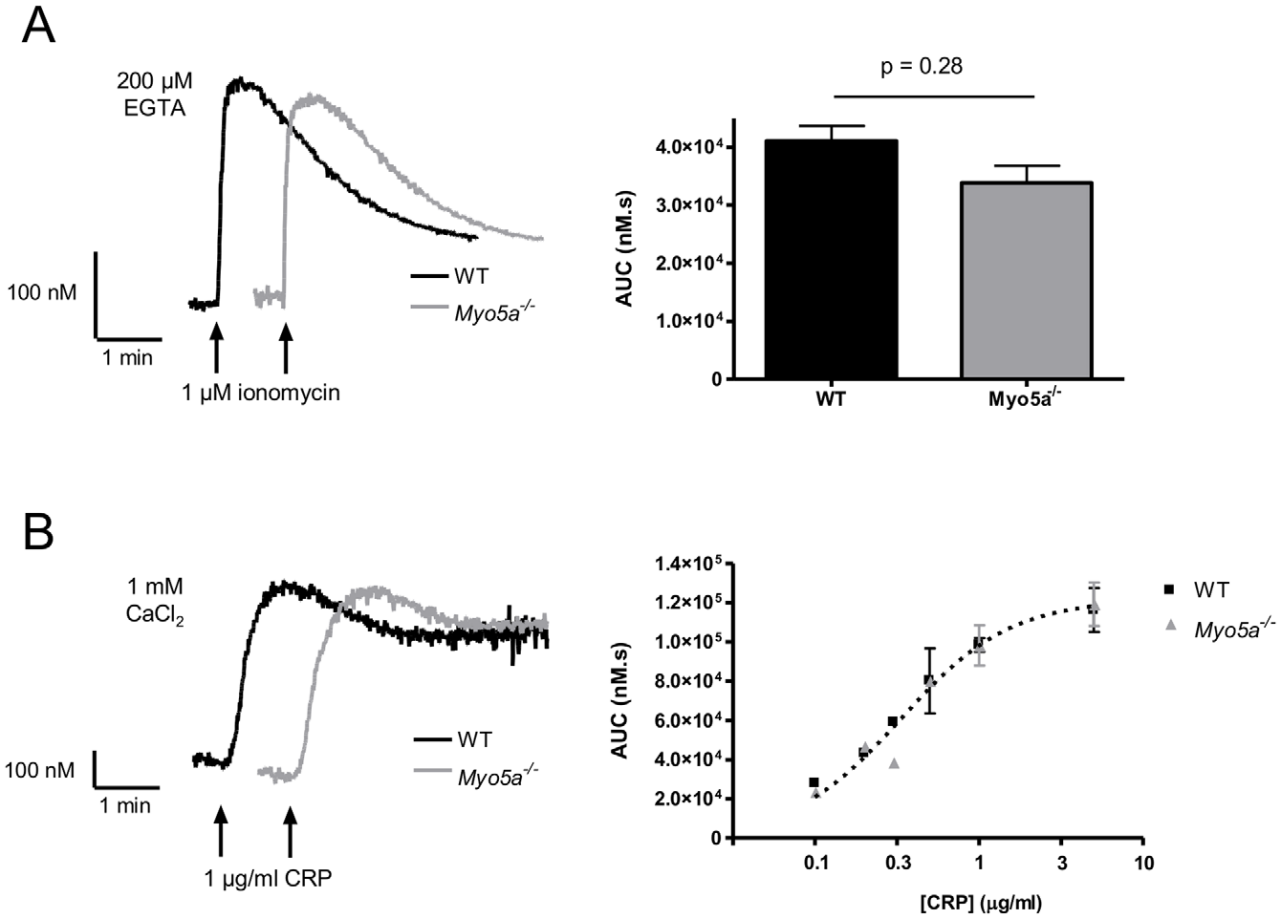
Normal Ca^2+^ signalling in *Myo5a* ^−/−^ platelets. Wild-type and *Myo5a* ^−/−^ platelets were loaded with the Ca^2+^-sensitive dye Fura-PE3. (A) Ionomycin (1 µM) was added in presence of the calcium chelator EGTA (200 µM). (B) Platelets were stimulated with the indicated concentrations of CRP in the presence of 1 mM CaCl_2_. The left hand panels show representative traces (n = 3). Data in the right hand panels (mean +/− SEM, n = 3) are expressed as area under the curve (AUC).

Myosin Va transports extensions of the ER into dendritic spines of Purkinje neurons, forming a local Ca^2+^ store that is required for local Ca^2+^ release [26]. We tested therefore whether myosin Va also transports Ca^2+^ stores into platelets during thrombopoiesis. Mouse platelets were loaded with the Ca^2+^-sensitive dye, Fura-PE3. Rapid depletion of the intracellular Ca^2+^ stores by ionomycin produced a transient increase in [Ca^2+^]_i_ that was not significantly different in *Myo5a* ^−/−^ platelets (Fig. 6A). Furthermore, Ca^2+^ signals in response to a range of concentrations of CRP were not significantly altered (Fig. 6B). These data suggest that myosin Va is not required to transport intracellular Ca^2+^ stores into platelets, since the stores are equivalently loaded with releasable calcium, and is not involved in agonist-induced Ca^2+^ signalling in platelets.

**Figure 7:**
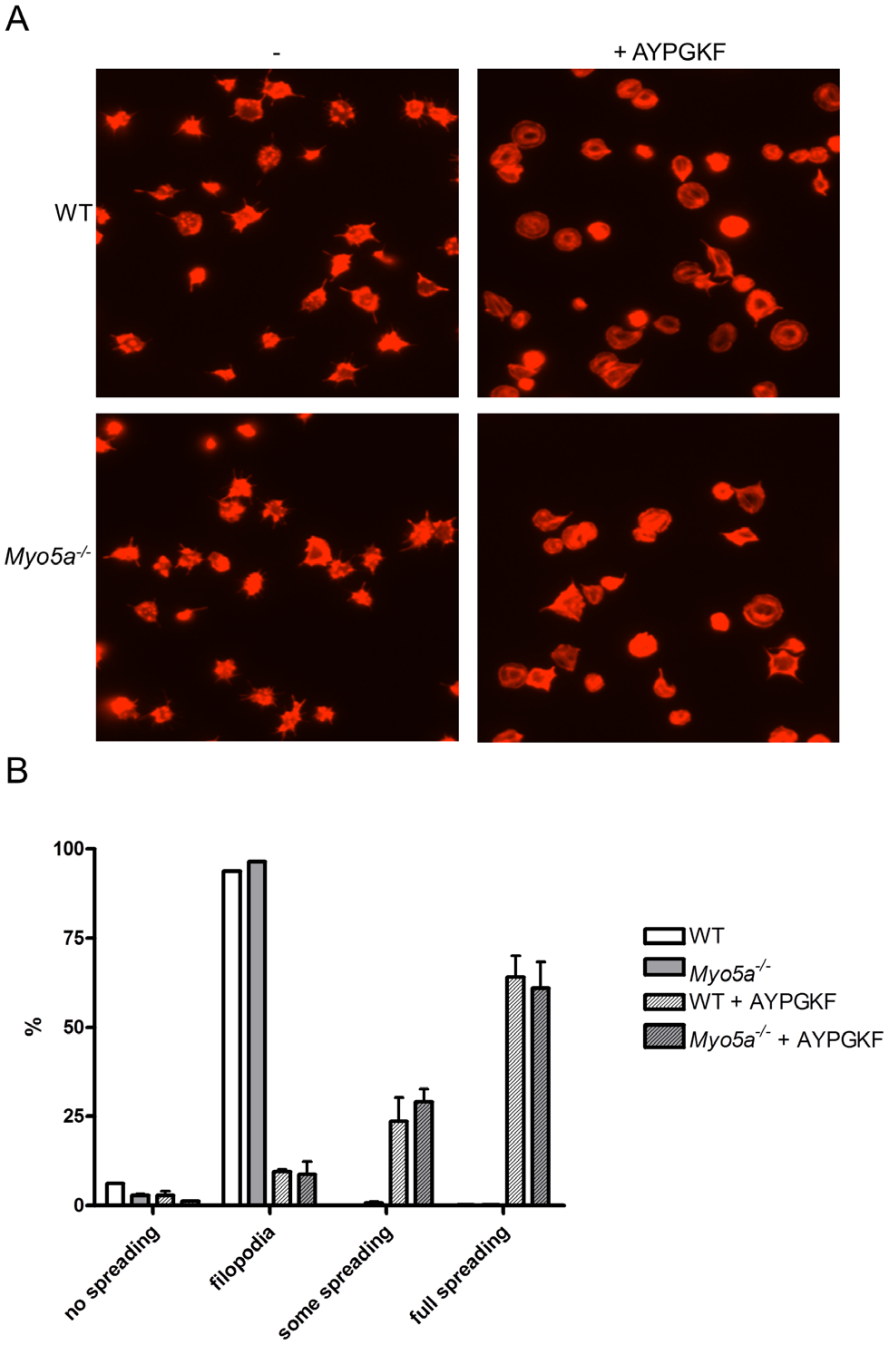
Loss of myosin Va does not affect platelet spreading on fibrinogen. Wild-type and *Myo5a* ^−/−^ mouse platelets were dispensed onto fibrinogen-coated coverslips for 1 h. Where indicated, platelets were stimulated with the PAR4 agonist AYPGKF (300 µM) for 1 min prior to adhesion. Cells were stained with TRITC-phalloidin. In (A), representative images are shown (n = 3). (B) Platelet morphological subtypes were counted and fractions (mean +/− SEM, n = 3) were calculated.

Finally, we analysed whether loss of myosin Va affects platelet spreading on a fibrinogen-coated surface. *Myo5a* ^−/−^ platelets did not show a difference in spreading on this surface whether in presence or absence of stimulation with AYPGKF (Fig. 7A). Morphological analysis also did not reveal any difference in subtype fractions (no spreading, filopodial, some spreading or full spreading) between myosin Va-deficient and wild-type platelets (Fig. 7B).

We conclude therefore that although myosin Va has been shown to be critical in intracellular actin-dependent vesicle transport in melanocytes and neuroendocrine cells, our data demonstrate that this motor protein is not required for mouse platelet granule secretion and other aspects of mouse platelet function. This contrasts to the important role for Rab27a/b in platelet dense granule biogenesis and secretion [9]–[11], and suggests that Rab27 proteins act through effectors other than myosin Va. One such effector is Munc13-4, which is essential for platelet dense granule secretion [27]–[28]. However, since Munc13-4-deficient platelets have normal granule numbers [27], there is likely to be another Rab27 effector that is involved in granule biogenesis that remains to be described.
